# 1,1′,2,2′-Tetra­methyl-3,3′-(*p*-phenyl­enedimethyl­ene)diimidazol-1-ium bis­(trifluoro­methane­sulfonate)

**DOI:** 10.1107/S1600536810033490

**Published:** 2010-08-28

**Authors:** Puvaneswary Subramaniam, Yatimah Alias, Kumuthini Chandrasekaram

**Affiliations:** a Department of Chemistry, University of Malaya, 50603 Kuala Lumpur, Malaysia

## Abstract

In the solid form of the title imidazolium-based ionic liquid salt, C_18_H_24_N_4_^2+^·2CF_3_SO_3_^−^, the complete cation is generated by a crystallographic inversion centre. The five-membered imidazole ring is approximately perpendicular to the six-membered phenyl­ene ring [dihedral angle = 85.11 (11)°]. In the crystal, the components are linked by C—H⋯O interactions.

## Related literature

For background to imidazolium-based ionic liquid salts, see: Ganesan *et al.* (2008 ▶); Puvaneswary *et al.* (2009*a* ▶,*b* ▶,*c* ▶). 
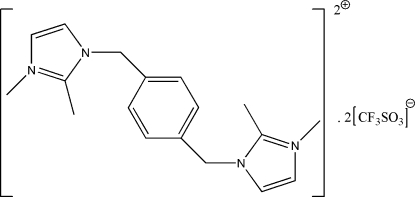


## Experimental

### 

#### Crystal data

C_18_H_24_N_4_^2+^·2CF_3_O_3_S^−^
*M* *_r_* = 594.55
Triclinic, 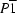

*a* = 7.3054 (1) Å
*b* = 8.0645 (2) Å
*c* = 11.3548 (2) Å
α = 89.946 (1)°
β = 76.653 (1)°
γ = 75.213 (1)°
*V* = 628.14 (2) Å^3^
*Z* = 1
Mo *K*α radiation
μ = 0.30 mm^−1^
*T* = 100 K
0.31 × 0.19 × 0.04 mm

#### Data collection

Bruker APEXII CCD diffractometer
Absorption correction: multi-scan (*SADABS*; Sheldrick, 1996 ▶) *T*_min_ = 0.912, *T*_max_ = 0.988
5105 measured reflections
2273 independent reflections
1982 reflections with *I* > 2σ(*I*)
*R*_int_ = 0.021

#### Refinement

*R*[*F* ^2^ > 2σ(*F* ^2^)] = 0.042
*wR*(*F* ^2^) = 0.103
*S* = 1.16
2273 reflections
174 parameters
H-atom parameters constrained
Δρ_max_ = 0.33 e Å^−3^
Δρ_min_ = −0.37 e Å^−3^

### 

Data collection: *APEX2* (Bruker, 2008 ▶); cell refinement: *SAINT* (Bruker, 2008 ▶); data reduction: *SAINT*; program(s) used to solve structure: *SHELXS97* (Sheldrick, 2008 ▶); program(s) used to refine structure: *SHELXL97* (Sheldrick, 2008 ▶); molecular graphics: *X-SEED* (Barbour, 2001 ▶); software used to prepare material for publication: *publCIF* (Westrip, 2010 ▶).

## Supplementary Material

Crystal structure: contains datablocks I, global. DOI: 10.1107/S1600536810033490/hg2702sup1.cif

Structure factors: contains datablocks I. DOI: 10.1107/S1600536810033490/hg2702Isup2.hkl

Additional supplementary materials:  crystallographic information; 3D view; checkCIF report

## Figures and Tables

**Table 1:** Hydrogen-bond geometry (Å, °)

*D*—H⋯*A*	*D*—H	H⋯*A*	*D*⋯*A*	*D*—H⋯*A*
C6—H6*A*⋯O2^i^	0.99	2.35	3.251 (3)	152
C3—H3*A*⋯O2^ii^	0.98	2.59	3.424 (4)	144
C1—H1*C*⋯O2^ii^	0.98	2.49	3.296 (3)	139
C1—H1*B*⋯O1^iii^	0.98	2.36	3.293 (3)	159
C1—H1*A*⋯O1^iv^	0.98	2.52	3.164 (3)	123

Symmetry codes: (i) 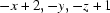; (ii) 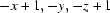; (iii) 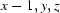; (iv) 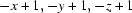.

**Fig. 1.**
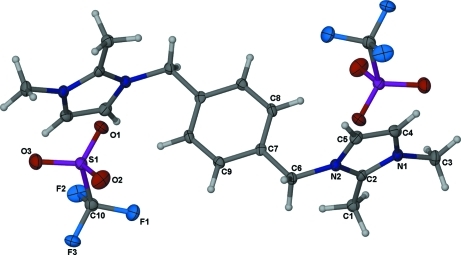
Thermal ellipsoid plot of the title compund at the 50% probability level. H atoms are drawn as spheres of arbitrary radius.

## supplementary crystallographic information

### Comment

Crystal structures of similar symmetrical compunds have been reported from our previous studies (Ganesan, *et al.*, 2008; Puvaneswary *et al.*, 2009*a*; 2009*b*; 2009*c*). As part of our ongoing research into imidazolium-based ionic liquids, we have synthesized 1,1',2,2'-tetramethyl-3,3'-(*p*-phenylenedimethylene)diimidazolium salt with trifluoromethanesulfonate anion.

The neighbouring cations in the title compund are held together *via* C–H···O hydrogen bonds in opposite directions through imidazole rings to CF_3_SO_3_^- ^anions and these hydrogen bonds stabilize the crystal structure.

### Experimental

α,α-Dibromo-*p*-xylene (1.26 g, 4.77 mmol) and 1,2-dimethylimidazole (0.96 g, 9.99 mmol) were refluxed in DMF (50 ml) for 3 h. The product that separated from solution was collected and washed with ether. Crystals of the bromide salt were grown from its solution in water (Puvaneswary *et al.*, 2009*a*).

The bromide salt (0.50 g, 1.10 mmol) and lithium trifluoromethanesulfonate (0.36 g, 2.31 mmol) were stirred in water (100 ml) for 24 h. Colourless crystals were obtained by slow evaporation of the solution (Melting point: 92–94°C).

### Refinement

Carbon-bound H-atoms were placed in calculated positions (C—H 0.95 to 0.99Å)and were included in the refinement in the riding model approximation, with *U*(H) set to 1.2 to 1.5*U*_eq_(C).

### Crystal data

**Table:**

C_18_H_24_N_4_^2+^·2CF_3_O_3_S^−^	*Z* = 1
*M**_r_* = 594.55	*F*(000) = 306
Triclinic, *P*1	*D*_x_ = 1.572 Mg m^−^^3^
Hall symbol: -P 1	Mo *K*α radiation, λ = 0.71073 Å
*a* = 7.3054 (1) Å	Cell parameters from 1992 reflections
*b* = 8.0645 (2) Å	θ = 2.6–29.0°
*c* = 11.3548 (2) Å	µ = 0.30 mm^−^^1^
α = 89.946 (1)°	*T* = 100 K
β = 76.653 (1)°	Plate, colourless
γ = 75.213 (1)°	0.31 × 0.19 × 0.04 mm
*V* = 628.14 (2) Å^3^	

### Data collection

**Table:**

Bruker APEXII CCD diffractometer	2273 independent reflections
Radiation source: fine-focus sealed tube	1982 reflections with *I* > 2σ(*I*)
graphite	*R*_int_ = 0.021
φ and ω scans	θ_max_ = 25.3°, θ_min_ = 1.9°
Absorption correction: multi-scan (*SADABS*; Sheldrick, 1996)	*h* = −8→8
*T*_min_ = 0.912, *T*_max_ = 0.988	*k* = −9→9
5105 measured reflections	*l* = −13→13

### Refinement

**Table:**

Refinement on *F*^2^	Primary atom site location: structure-invariant direct methods
Least-squares matrix: full	Secondary atom site location: difference Fourier map
*R*[*F*^2^ > 2σ(*F*^2^)] = 0.042	Hydrogen site location: inferred from neighbouring sites
*wR*(*F*^2^) = 0.103	H-atom parameters constrained
*S* = 1.16	*w* = 1/[σ^2^(*F*_o_^2^) + (0.0241*P*)^2^ + 1.0056*P*] where *P* = (*F*_o_^2^ + 2*F*_c_^2^)/3
2273 reflections	(Δ/σ)_max_ < 0.001
174 parameters	Δρ_max_ = 0.33 e Å^−^^3^
0 restraints	Δρ_min_ = −0.37 e Å^−^^3^

### Special details

**Table:**

Geometry. All e.s.d.'s (except the e.s.d. in the dihedral angle between two l.s. planes) are estimated using the full covariance matrix. The cell e.s.d.'s are taken into account individually in the estimation of e.s.d.'s in distances, angles and torsion angles; correlations between e.s.d.'s in cell parameters are only used when they are defined by crystal symmetry. An approximate (isotropic) treatment of cell e.s.d.'s is used for estimating e.s.d.'s involving l.s. planes.
Refinement. Refinement of *F*^2^ against ALL reflections. The weighted *R*-factor *wR* and goodness of fit *S* are based on *F*^2^, conventional *R*-factors *R* are based on *F*, with *F* set to zero for negative *F*^2^. The threshold expression of *F*^2^ > σ(*F*^2^) is used only for calculating *R*-factors(gt) *etc*. and is not relevant to the choice of reflections for refinement. *R*-factors based on *F*^2^ are statistically about twice as large as those based on *F*, and *R*- factors based on ALL data will be even larger.

### Fractional atomic coordinates and isotropic or equivalent isotropic displacement parameters (Å^2^)

**Table:**

	*x*	*y*	*z*	*U*_iso_*/*U*_eq_	
N1	0.1441 (3)	0.2762 (3)	0.18655 (19)	0.0160 (5)	
N2	0.3402 (3)	0.2113 (3)	0.30441 (19)	0.0154 (5)	
C1	−0.0172 (4)	0.3018 (3)	0.4080 (2)	0.0184 (6)	
H1A	−0.1168	0.4026	0.3949	0.028*	
H1B	0.0251	0.3241	0.4810	0.028*	
H1C	−0.0718	0.2022	0.4184	0.028*	
C2	0.1513 (4)	0.2664 (3)	0.3023 (2)	0.0153 (5)	
C3	−0.0369 (4)	0.3301 (4)	0.1445 (3)	0.0214 (6)	
H3A	−0.1169	0.2498	0.1703	0.032*	
H3B	−0.0050	0.3304	0.0558	0.032*	
H3C	−0.1093	0.4459	0.1793	0.032*	
C4	0.3304 (4)	0.2260 (3)	0.1129 (2)	0.0192 (6)	
H4	0.3651	0.2214	0.0268	0.023*	
C5	0.4525 (4)	0.1851 (3)	0.1863 (2)	0.0191 (6)	
H5	0.5906	0.1454	0.1620	0.023*	
C6	0.4164 (4)	0.1924 (3)	0.4137 (2)	0.0165 (6)	
H6A	0.5389	0.0992	0.3970	0.020*	
H6B	0.3214	0.1575	0.4796	0.020*	
C7	0.4564 (3)	0.3550 (3)	0.4568 (2)	0.0144 (5)	
C8	0.4553 (4)	0.4977 (3)	0.3878 (2)	0.0166 (6)	
H8	0.4247	0.4974	0.3109	0.020*	
C9	0.5011 (4)	0.3589 (3)	0.5692 (2)	0.0155 (5)	
H9	0.5018	0.2624	0.6173	0.019*	
S1	1.07181 (10)	0.18928 (9)	0.76648 (6)	0.01802 (18)	
F1	0.7150 (2)	0.1636 (2)	0.78601 (16)	0.0331 (4)	
F2	0.7393 (2)	0.3543 (2)	0.91048 (16)	0.0334 (4)	
F3	0.8292 (2)	0.0858 (2)	0.94182 (14)	0.0272 (4)	
O1	1.0372 (3)	0.3265 (3)	0.68688 (17)	0.0283 (5)	
O2	1.1362 (3)	0.0188 (3)	0.70802 (19)	0.0332 (5)	
O3	1.1741 (3)	0.2164 (3)	0.85592 (18)	0.0262 (5)	
C10	0.8268 (4)	0.1991 (3)	0.8556 (2)	0.0204 (6)	

### Atomic displacement parameters (Å^2^)

**Table:**

	*U*^11^	*U*^22^	*U*^33^	*U*^12^	*U*^13^	*U*^23^
N1	0.0189 (12)	0.0156 (11)	0.0150 (11)	−0.0052 (9)	−0.0060 (9)	−0.0006 (9)
N2	0.0154 (11)	0.0174 (12)	0.0136 (11)	−0.0046 (9)	−0.0035 (9)	0.0001 (9)
C1	0.0180 (14)	0.0191 (14)	0.0177 (13)	−0.0044 (11)	−0.0039 (11)	0.0009 (11)
C2	0.0188 (13)	0.0130 (13)	0.0162 (13)	−0.0063 (11)	−0.0059 (11)	0.0002 (10)
C3	0.0221 (15)	0.0233 (15)	0.0215 (14)	−0.0053 (12)	−0.0111 (12)	0.0003 (11)
C4	0.0247 (15)	0.0190 (14)	0.0136 (13)	−0.0081 (12)	−0.0009 (11)	−0.0017 (10)
C5	0.0173 (14)	0.0215 (14)	0.0174 (14)	−0.0062 (11)	−0.0007 (11)	−0.0032 (11)
C6	0.0160 (13)	0.0192 (14)	0.0157 (13)	−0.0050 (11)	−0.0060 (10)	0.0020 (10)
C7	0.0073 (12)	0.0192 (13)	0.0151 (13)	−0.0022 (10)	−0.0011 (10)	−0.0011 (10)
C8	0.0150 (13)	0.0227 (14)	0.0123 (12)	−0.0038 (11)	−0.0047 (10)	0.0009 (10)
C9	0.0131 (13)	0.0171 (13)	0.0155 (13)	−0.0022 (10)	−0.0041 (10)	0.0029 (10)
S1	0.0180 (4)	0.0213 (4)	0.0143 (3)	−0.0061 (3)	−0.0019 (3)	−0.0007 (3)
F1	0.0283 (10)	0.0448 (11)	0.0367 (10)	−0.0189 (8)	−0.0182 (8)	0.0115 (8)
F2	0.0254 (9)	0.0241 (9)	0.0409 (11)	0.0004 (7)	0.0038 (8)	−0.0048 (8)
F3	0.0329 (10)	0.0300 (9)	0.0213 (9)	−0.0150 (8)	−0.0044 (7)	0.0088 (7)
O1	0.0382 (12)	0.0340 (12)	0.0190 (10)	−0.0182 (10)	−0.0098 (9)	0.0098 (9)
O2	0.0293 (12)	0.0287 (12)	0.0355 (13)	−0.0059 (9)	0.0024 (10)	−0.0137 (10)
O3	0.0222 (11)	0.0357 (12)	0.0227 (11)	−0.0089 (9)	−0.0081 (8)	0.0034 (9)
C10	0.0216 (15)	0.0187 (14)	0.0209 (14)	−0.0047 (11)	−0.0060 (12)	0.0023 (11)

### Geometric parameters (Å, °)

**Table:**

N1—C2	1.329 (3)	C6—C7	1.520 (4)
N1—C4	1.382 (3)	C6—H6A	0.9900
N1—C3	1.471 (3)	C6—H6B	0.9900
N2—C2	1.343 (3)	C7—C9	1.390 (4)
N2—C5	1.386 (3)	C7—C8	1.391 (4)
N2—C6	1.466 (3)	C8—C9^i^	1.392 (4)
C1—C2	1.475 (4)	C8—H8	0.9500
C1—H1A	0.9800	C9—C8^i^	1.392 (4)
C1—H1B	0.9800	C9—H9	0.9500
C1—H1C	0.9800	S1—O1	1.438 (2)
C3—H3A	0.9800	S1—O3	1.439 (2)
C3—H3B	0.9800	S1—O2	1.442 (2)
C3—H3C	0.9800	S1—C10	1.824 (3)
C4—C5	1.339 (4)	F1—C10	1.338 (3)
C4—H4	0.9500	F2—C10	1.331 (3)
C5—H5	0.9500	F3—C10	1.338 (3)
			
C2—N1—C4	109.9 (2)	N2—C6—C7	113.6 (2)
C2—N1—C3	124.5 (2)	N2—C6—H6A	108.8
C4—N1—C3	125.6 (2)	C7—C6—H6A	108.8
C2—N2—C5	109.0 (2)	N2—C6—H6B	108.8
C2—N2—C6	125.6 (2)	C7—C6—H6B	108.8
C5—N2—C6	125.3 (2)	H6A—C6—H6B	107.7
C2—C1—H1A	109.5	C9—C7—C8	118.8 (2)
C2—C1—H1B	109.5	C9—C7—C6	118.2 (2)
H1A—C1—H1B	109.5	C8—C7—C6	123.0 (2)
C2—C1—H1C	109.5	C7—C8—C9^i^	120.3 (2)
H1A—C1—H1C	109.5	C7—C8—H8	119.8
H1B—C1—H1C	109.5	C9^i^—C8—H8	119.8
N1—C2—N2	107.0 (2)	C7—C9—C8^i^	120.9 (2)
N1—C2—C1	126.3 (2)	C7—C9—H9	119.5
N2—C2—C1	126.6 (2)	C8^i^—C9—H9	119.5
N1—C3—H3A	109.5	O1—S1—O3	115.23 (12)
N1—C3—H3B	109.5	O1—S1—O2	115.05 (13)
H3A—C3—H3B	109.5	O3—S1—O2	114.61 (13)
N1—C3—H3C	109.5	O1—S1—C10	102.57 (13)
H3A—C3—H3C	109.5	O3—S1—C10	103.71 (12)
H3B—C3—H3C	109.5	O2—S1—C10	103.25 (13)
C5—C4—N1	106.8 (2)	F2—C10—F3	107.4 (2)
C5—C4—H4	126.6	F2—C10—F1	107.8 (2)
N1—C4—H4	126.6	F3—C10—F1	107.1 (2)
C4—C5—N2	107.3 (2)	F2—C10—S1	111.33 (19)
C4—C5—H5	126.4	F3—C10—S1	111.72 (19)
N2—C5—H5	126.4	F1—C10—S1	111.34 (19)

Symmetry codes: (i) −*x*+1, −*y*+1, −*z*+1.

### Hydrogen-bond geometry (Å, °)

**Table:**

*D*—H···*A*	*D*—H	H···*A*	*D*···*A*	*D*—H···*A*
C6—H6A···O2^ii^	0.99	2.35	3.251 (3)	152.
C3—H3A···O2^iii^	0.98	2.59	3.424 (4)	144.
C1—H1C···O2^iii^	0.98	2.49	3.296 (3)	139.
C1—H1B···O1^iv^	0.98	2.36	3.293 (3)	159.
C1—H1A···O1^i^	0.98	2.52	3.164 (3)	123.

Symmetry codes: (ii) −*x*+2, −*y*, −*z*+1; (iii) −*x*+1, −*y*, −*z*+1; (iv) *x*−1, *y*, *z*; (i) −*x*+1, −*y*+1, −*z*+1.
